# Early Pregnancy Termination with Mifepristone and Misoprostol: Concurrent vs. 48-Hour Interval Administration in a Randomized Controlled Trial

**DOI:** 10.3390/jcm14217616

**Published:** 2025-10-27

**Authors:** Meirav Braverman, Adi Dayan-Schwartz, Yehuda Ben-David, Orly Kachta, Noah Zafran

**Affiliations:** 1 Department of Obstetrics and Gynecology, Emek Medical Center, Afula 1834111, Israel; 2 Rappaport Faculty of Medicine, Technion-Israel Institute of Technology, Haifa 3103301, Israel

**Keywords:** medical termination of pregnancy, mifepristone, misoprostol, first-trimester termination, retained products of conception

## Abstract

**Background**: The standard protocol for early first-trimester termination of pregnancy (TOP) involves administration of mifepristone followed by misoprostol after a 48-h interval. While concurrent administration may improve convenience and access, evidence regarding its effectiveness remains limited. This study aims to compare the efficacy, safety, and acceptability of concurrent oral administration of mifepristone and misoprostol with the 48-h interval regimen for early TOP. **Methods**: In this randomized controlled trial (ClinicalTrials.gov: NCT03440866), 250 patients with intrauterine pregnancies up to 49 days’ gestation were randomized to receive either concurrent treatment (600 mg mifepristone and 400 mcg misoprostol) or the same medications administered 48 h apart. The primary outcome was complete abortion without additional intervention. Secondary outcomes included adverse events, pain, and patient satisfaction. Follow-up occurred approximately two weeks post-treatment. Data were available for 220 participants. **Results**: The concurrent group had a significantly lower success rate compared to the control group (68.8% vs. 84.3%, *p* = 0.007). Continuing pregnancy was more frequent with concurrent administration (13.4% vs. 2.8%, *p* = 0.004). No significant differences were observed in hemoglobin change, adverse events, or pain scores. Patient satisfaction was higher in the control group (81.1% vs. 63.6%, *p* = 0.04), though preferences for future abortion methods did not differ between groups. **Conclusions**: Concurrent administration of mifepristone and misoprostol is less effective and less satisfactory than the standard 48-h regimen, although safety and pain profiles are comparable. It should not replace the interval protocol, and patients choosing concurrent treatment should be counseled about its lower efficacy and higher likelihood of requiring additional intervention.

## 1. Introduction

Termination of pregnancy (TOP) is among the most frequently performed medical procedures worldwide and represents an essential component of comprehensive reproductive healthcare. In the United States alone, the estimated TOP rate in 2022 was 11.2 per 1000 women of reproductive age, and more than half of these procedures—approximately 57.6%—were achieved through medical rather than surgical means [1]. The steady increase in the proportion of medical abortions reflects a larger global pattern and illustrates how medication-based approaches have become firmly embedded in contemporary obstetric and gynecologic practice.

Medical abortion regimens have been widely endorsed and incorporated into official clinical recommendations by leading international professional bodies, including the World Health Organization (WHO, Geneva, Switzerland) and the American College of Obstetricians and Gynecologists (ACOG, Washington, DC, USA) [2,3]. These evidence-based protocols emphasize the combined use of two agents, mifepristone and misoprostol, rather than misoprostol alone. Widespread implementation of this regimen has been credited with reducing unsafe abortions and related maternal morbidity and mortality by providing a safer alternative where surgical services are limited. Numerous clinical trials and meta-analyses have demonstrated that the combined regimen produces higher rates of complete abortion and reduces the likelihood of requiring surgical evacuation compared with single-agent therapy [4,5,6]. This dual-drug strategy has therefore become the standard of care in many healthcare systems.

On a global level, the use of medication abortion has increased steadily over the past several decades [7,8]. This shift reflects not only evolving clinical evidence but also changing patient expectations and social preferences. For many patients, a medication-induced miscarriage offers an important alternative to surgical evacuation because it allows the process to occur in the privacy and comfort of one’s own home, often with less disruption to daily life. In contrast, surgical abortion typically requires attendance at a healthcare facility, exposure to anesthesia, and, in some cases, longer recovery periods [9]. When patients are given the option to choose between medical and surgical termination, their preferences are shaped by a complex interplay of factors, including anticipated or experienced pain, the total duration of the process, and the need for repeated clinical encounters [7]. Most physicians regard both approaches as acceptable, but individual circumstances—such as personal medical history, logistical considerations, or cultural environment—often determine which option is ultimately chosen. This underscores the importance of ensuring that both modalities remain available and that patients are fully informed about their respective advantages and limitations.

The pharmacological underpinnings of medical abortion rely on the complementary actions of mifepristone and misoprostol [2,3]. Mifepristone is a potent progesterone receptor antagonist. It was first introduced as a combined medical abortion regimen in the late 1980s [10]. Mifepristone was approved for use in France and China in 1988, and its adoption has since expanded worldwide; as of 2023, it is approved in 96 countries [11]. By blocking progesterone, which is essential for maintaining early pregnancy, it induces decidual necrosis, softens the cervix, and increases the contractility of the uterus through enhanced sensitivity to prostaglandins [12]. Progesterone withdrawal triggered by mifepristone initiates a cascade of physiological and biochemical events that collectively prime the uterus and cervix for expulsion. Research has shown that mifepristone stimulates the release of endogenous prostaglandins and significantly enhances myometrial responsiveness to both prostaglandins and oxytocin [13,14]. At the cervical level, it promotes nitric oxide production and upregulates inducible nitric oxide synthase, which contributes to cervical softening and dilation [15]. Additional pathways involve increased expression of oxytocin and prostaglandin receptors, further facilitating the process of cervical ripening [16]. Taken together, these mechanisms optimize the environment for the subsequent administration of misoprostol.

Misoprostol, a synthetic prostaglandin E1 analog, is valued in this context for several reasons. It is inexpensive, widely available, chemically stable at room temperature, and can be administered by a variety of routes [17]. Misoprostol’s pharmacokinetic profile depends on its route of administration. It can be given orally, buccally (absorbed in the cheek), sublingually (under the tongue), or vaginally, and each route has implications for both efficacy and side-effect profiles. Vaginal administration, which leads to slower but more sustained uterine exposure, has consistently been associated with high efficacy and lower rates of incomplete abortion [18]. Buccal and sublingual routes are nearly as effective as the vaginal route and often act more rapidly, but their higher systemic absorption can produce increased gastrointestinal side effects, including diarrhea, chills, and feverish sensations [19]. Oral misoprostol, while still effective in many cases, has lower bioavailability and a shorter duration of action, leading to slightly lower overall success rates. Current guidelines, including those issued by WHO, emphasize flexibility in route selection, allowing patients and providers to tailor administration to individual preferences, tolerability, and context—whether in hospital settings, outpatient clinics, or at home [20]. This patient-centered approach highlights the balance between optimizing pharmacological efficacy and respecting patient autonomy and comfort.

The original regimen approved by the U.S. Food and Drug Administration (FDA) specified 600 mg of oral mifepristone followed 48 h later by 400 mcg of oral misoprostol. This protocol achieved complete abortion rates of approximately 87–92% in early studies [21,22]. The rationale for the 36–48-h interval between the two agents was based on experimental and clinical evidence showing that this delay maximizes uterine sensitivity to prostaglandins and increases the likelihood of complete expulsion [23,24,25]. While highly effective, the requirement for a return clinic visit and the imposed waiting period have been viewed as potential barriers to access for some patients.

Efforts to modify this interval, including shortening the delay or administering the medications concurrently, aim to simplify the treatment process, improve patient convenience, and reduce the number of necessary clinic visits. Such adjustments could be particularly valuable in settings where healthcare access is limited, where multiple visits pose logistical challenges, or during periods of restricted healthcare mobility, such as the COVID-19 pandemic. However, the available data regarding the efficacy of concurrent administration remains limited. Although several prior studies have investigated alternative dosing strategies and shortened intervals [26,27], none had directly compared concurrent administration of 600 mg oral mifepristone with 400 mcg oral misoprostol against the standard 48-h interval protocol at the time of our trial.

To address this critical gap in the literature, we conducted a randomized controlled trial to compare the efficacy and safety of concurrent versus 48-h interval administration of mifepristone and misoprostol for early medical termination of pregnancy.

## 2. Materials and Methods

The study was designed as a prospective, randomized, open-label controlled clinical trial. It was carried out at a large university-affiliated hospital located in Afula, Israel, over a 16-month period between July 2018 and November 2019. The hospital serves as a regional referral center, ensuring a diverse patient population that includes both urban and rural communities. The trial was conducted in accordance with the principles of the Declaration of Helsinki. Approval was obtained from the Institutional Review Board (IRB approval number 0002-17-EMC) prior to initiation, and all participants provided written informed consent before enrollment.

Patients were considered eligible if they were aged 18 years or older, had a confirmed intrauterine pregnancy by transvaginal ultrasound, and were seeking termination of pregnancy at a gestational age up to and including 49 days. Approval for pregnancy termination was granted in accordance with Israeli law and national guidelines [28].

Exclusion criteria were carefully defined to protect patient safety and to avoid potential confounders. Patients were excluded if they had an ectopic pregnancy, an intrauterine device (IUD) in situ, chronic systemic steroid use, known adrenal insufficiency, coagulopathy, ongoing anticoagulant therapy, acute or chronic porphyria, known drug hypersensitivity, or significant systemic illness such as uncontrolled hypertension, renal or hepatic impairment, severe asthma, or anemia with hemoglobin levels below 9 g/dL. Breastfeeding patients were also excluded because of concerns regarding drug transfer into breast milk. These strict inclusion and exclusion criteria were chosen to ensure a homogeneous population suitable for comparison and to minimize avoidable risks.

Participants were randomized in a 1:1 ratio to one of the two study arms using a computer-generated randomization sequence. Allocation concealment was ensured by the use of sequentially numbered, sealed envelopes prepared by personnel not directly involved in patient care, opened only after patient enrollment. This method minimized selection bias and ensured equal distribution of participants between groups. Blinding of participants and clinical staff was not feasible due to the nature of the intervention and the distinct timeframes between drug administrations; however, the subsequent statistical analysis of outcomes was performed by reviewers blinded to the allocation, reducing the risk of analytical bias.

Patient confidentiality was strictly maintained throughout the trial. Upon enrollment, each participant was assigned a unique study ID, and all data were recorded under this code without identifying information. Only authorized study personnel had access to the encrypted data files.

Concurrent group: Participants randomized to this arm received 600 mg of oral mifepristone followed immediately by 400 mcg of oral misoprostol. Both medications were administered under direct observation in the hospital setting. Patients remained in the ward for at least three hours of observation to monitor for immediate adverse events such as excessive bleeding, severe abdominal pain, or allergic reactions.

Interval group: Participants in the control arm received 600 mg of oral mifepristone, also under supervision, followed 48 h later by 400 mcg of oral misoprostol. After misoprostol administration, patients were observed for a minimum of two hours. This observation period ensured uniformity in monitoring and allowed clinical staff to provide supportive care if needed.

The oral route of misoprostol administration and the dosage used were in accordance with the original U.S. FDA-approved regimen for medical termination of pregnancy up to 49 days of gestation [29].

All participants received standard supportive care during the abortion process. Supportive measures, such as the provision of analgesics (non-steroidal anti-inflammatory drugs or acetaminophen) and antiemetics, were offered on demand according to patient preference. This approach is in line with WHO guidelines recommending routine pain relief during medical abortion [20]. Patients were advised on non-pharmacological comfort measures (rest in a quiet environment, positioning for comfort, relaxation, and encouragement of partner or companion presence when desired). Surgical backup was available on site throughout the study to manage potential complications.

Participants were instructed to return for follow-up approximately 14 days after treatment. Follow-up assessments included a comprehensive gynecological examination, a transvaginal ultrasound scan to confirm the absence of an ongoing pregnancy or retained products of conception, and hemoglobin testing. Pain intensity during the abortion process was assessed using a validated 0–10 Verbal Numerical Rating Scale (VNRS), where 0 indicated no pain and 10 represented the worst pain imaginable. Patient satisfaction was recorded using a 5-point Likert scale ranging from “very dissatisfied” to “very satisfied.” Additionally, participants were asked about their preferred method of termination should they require the procedure again in the future, providing insight into acceptability and patient-centered perspectives.

The patient satisfaction survey template is provided in the Supplementary Materials.

### 2.1. Outcome Measures

The primary outcome of the study was complete abortion, defined as termination of pregnancy without the need for any additional medical or surgical intervention.Secondary outcomes included: Incidence of excessive vaginal bleeding, changes in hemoglobin concentration before and after treatment, frequency and type of adverse events (e.g., infection, pelvic inflammatory disease, need for hospitalization), pain scores reported on the VNRS, overall patient satisfaction, and patient preference for future abortion methods.

Cases were classified as treatment failures if the patient experienced continuing pregnancy, incomplete abortion requiring further intervention, or excessive bleeding necessitating emergency treatment. Patients with suspected retained products of conception (RPOC) but without significant symptoms were monitored conservatively and referred for hysteroscopy or uterine evacuation only if findings persisted.

### 2.2. Sample Size

The sample size was calculated based on the assumption of a 97% [30] success rate for the standard regimen. To detect a 10% absolute difference in efficacy between the two groups with 80% statistical power and a two-sided alpha level of 0.05, a minimum of 115 patients per arm was required. Anticipating potential dropouts or loss to follow-up, we increased the target enrollment to 250 participants in total, ensuring adequate power for the primary outcome.

### 2.3. Statistical Analysis

Data analysis followed the principle of intention-to-treat, including all randomized participants in their originally assigned groups regardless of protocol adherence. Continuous variables such as age, gestational age, and hemoglobin levels were analyzed using Student’s t-test for normally distributed data or the Wilcoxon rank-sum test for skewed distributions. Categorical variables such as abortion success rates, adverse event frequencies, and satisfaction levels were compared using chi-square tests or Fisher’s exact test where appropriate. Missing data were handled using sensitivity analyses to evaluate potential impact. All analyses were conducted using SPSS version 23 (IBM, Chicago, IL, USA), and a two-tailed *p*-value < 0.05 was considered statistically significant.

The study was reported in accordance with the CONSORT 2025 guidelines, and the completed CONSORT 2025 Checklist is provided in the Supplementary Materials.

## 3. Results

**Figure 1:**
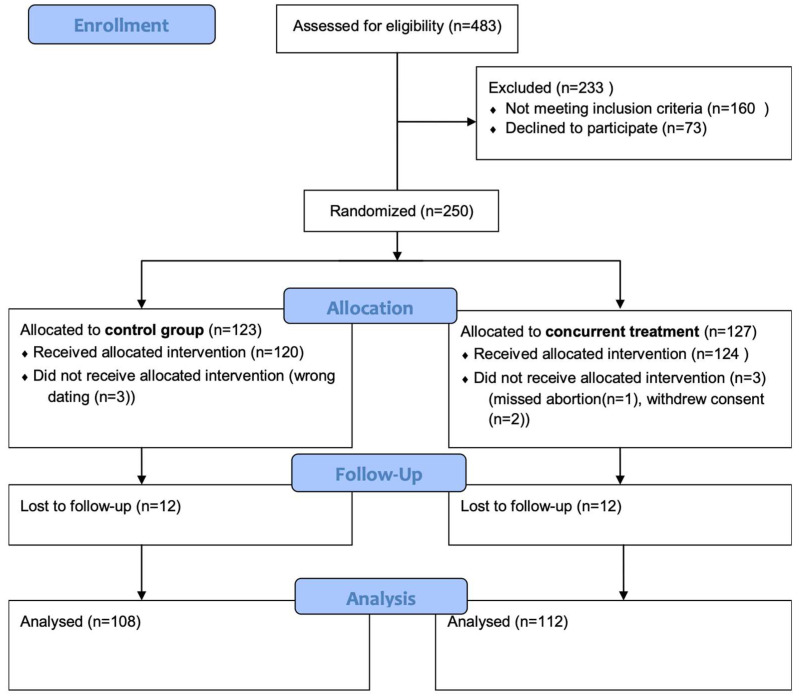
Flow diagram of the study population.

A total of 250 patients were enrolled in the study and provided informed consent for participation. After randomization, 127 patients were initially assigned to the concurrent treatment group, and 123 patients were allocated to the 48-h interval group. Six participants were later excluded from the analysis: four were found to be ineligible after randomization, and two withdrew consent prior to receiving treatment. This left 244 patients in the final study population who formed the basis of all subsequent analyses (Figure 1).

Baseline demographic and obstetric characteristics were similar between the two groups (Table 1). In both groups, the mean maternal age was approximately 32 years. Pre-treatment hemoglobin levels were also similar, with median values around 12.4 g/dL in both arms.

At baseline, all participants had confirmed intrauterine gestational sacs on ultrasound at enrollment. The mean gestational age, as determined by transvaginal ultrasound, was 6.0 ± 0.6 weeks, with values ranging from 4.4 to 7.0 weeks, and there was no meaningful difference between the two study arms. In addition, pre-treatment vaginal bleeding was reported by 9.1% of participants, again with no significant difference between the groups. These pregnancy characteristics are summarized in Table 2.

Follow-up data were available for 220 participants: 112 in the concurrent group and 108 in the interval group. This represented an overall follow-up rate of 90.2%. Among the study population, 52 patients (23.6%) required additional intervention, either surgical evacuation or an extra dose of misoprostol, while 47 patients (21.4%) ultimately underwent surgical procedures. Importantly, the proportion requiring any form of additional treatment was significantly higher in the concurrent group compared to the interval group (31.2% vs. 15.7%, *p* = 0.007). Similarly, continuing pregnancy occurred more frequently in the concurrent arm, affecting 13.4% of participants compared with only 2.8% in the interval arm (*p* = 0.004) (Table 3).

Within the concurrent group, 31 patients underwent surgical intervention. The indications were diverse: 15 procedures were performed due to continuing pregnancy, 6 due to excessive bleeding, and 3 for incomplete or missed abortion. In addition, 6 patients underwent hysteroscopy for RPOC, and one patient required intervention for pelvic inflammatory disease in association with RPOC. In contrast, only 15 patients in the interval group underwent surgical management: 3 for continuing pregnancy, 3 for excessive bleeding, and 9 for hysteroscopy due to RPOC.

Hemoglobin measurements were available for a substantial subset of patients without ongoing pregnancy—77 patients (68.8%) in the concurrent group and 82 patients (75.9%) in the interval group. Median post-treatment hemoglobin levels did not differ significantly between the groups. Similarly, the mean magnitude of hemoglobin decline was modest and comparable (0.20 g/dL in the concurrent group versus 0.30 g/dL in the interval group, *p* = 0.61). Notably, one patient in the concurrent group experienced a severe hemoglobin drop of 8.7 g/dL due to heavy bleeding, representing an outlier event that was carefully managed clinically.

Adverse events were infrequent overall and were similarly distributed between the two study arms (Table 4). Excessive bleeding occurred in a small minority of patients, and the frequency did not differ significantly. Emergency surgical evacuation for bleeding was required in 6 patients in the concurrent group and 3 patients in the interval group (*p*-value = 0.34), but this difference was not statistically significant. Blood transfusion was rare, occurring in only 2 patients, both in the concurrent group. Each arm recorded a single case of pelvic inflammatory disease. The patient in the concurrent arm required hospitalization and surgical intervention, whereas the patient in the interval arm was managed successfully with outpatient antibiotic therapy. The need for hysteroscopy for suspected RPOC was also similar between groups (5.4% in the concurrent arm vs. 8.3% in the interval arm, *p* = 0.38).

Pain scores, assessed using the VNRS, were similar between groups. Preferences for future abortion methods also did not differ significantly between treatment arms. However, satisfaction ratings revealed a clear difference. Significantly more patients in the interval group reported high levels of satisfaction, defined as a Likert score of 4 or above, compared to those in the concurrent group (81.1% vs. 63.6%, *p* = 0.008).

## 4. Discussion

This randomized controlled trial provides new evidence on the timing of mifepristone and misoprostol administration for early medical TOP. Our results demonstrate that concurrent administration of 600 mg mifepristone and 400 mcg misoprostol is significantly less effective than the standard 48-h interval regimen. Specifically, patients in the concurrent arm were more likely to experience treatment failure, defined as continuing pregnancy or incomplete abortion requiring additional medical or surgical intervention. Despite this important difference in efficacy, it is notable that the concurrent protocol was not associated with a higher rate of adverse events. In addition, patient-reported pain scores did not differ significantly between groups, suggesting that regimen timing primarily influenced efficacy rather than perceived physical burden.

Although both regimens can be considered clinically acceptable, the overall success rates we observed were somewhat lower than those reported in prior studies of medical abortion. Several factors may account for this discrepancy. First, our trial used an oral misoprostol regimen (according to the original U.S. FDA-approved regimen), which is known to have lower bioavailability compared with vaginal, buccal, or sublingual routes. Evidence from randomized studies and meta-analyses suggests that non-oral routes achieve higher tissue concentrations and sustain uterine activity for longer, thereby improving expulsion rates [31]. In addition, differences in protocol design, such as shorter dosing intervals [25], lower misoprostol dosages [32], and absence of repeat dosing [33]. Finally, the relatively short follow-up period in our study may have underestimated delayed expulsions that occur beyond two weeks [32]. Collectively, these methodological factors may explain the modestly lower success rates observed in both arms.

The oral route, despite its relatively lower systemic absorption compared to vaginal or sublingual administration, offers several practical advantages [34]: it has a shorter onset of action compared to the alternatives; it is non-invasive, simple to administer; and it requires no specialized equipment or privacy.

Despite the limitations, our findings highlight that even concurrent administration retains clear advantages over misoprostol monotherapy. The observed success rate of 68.8% in the concurrent group was substantially higher than historical rates for 400 mcg misoprostol alone [31]. This confirms that mifepristone exerts a beneficial priming effect on the uterus and cervix even when given simultaneously with misoprostol, underscoring the physiological synergy between these two agents.

Our results also provide reassurance that shortening the interval between mifepristone and misoprostol does not increase risks of hemorrhage or infection. Hemoglobin declines were comparable across groups, and serious complications were rare. This aligns with earlier observational studies suggesting that timing adjustments primarily influence expulsion efficacy rather than safety [24,33,35]. Likewise, patient-reported pain was similar across regimens, echoing prior reports that uterine cramping intensity is more closely related to misoprostol activity than to the timing of mifepristone priming.

Patient acceptability is another critical dimension of abortion care. Satisfaction scores in our study were significantly higher in the 48-h interval group, reflecting the higher success rate of that regimen. This finding is consistent with broader literature showing that satisfaction strongly correlates with treatment completeness and the avoidance of additional interventions [36]. Interestingly, however, preferences for future abortion method did not differ between the concurrent and interval groups, suggesting that even patients who experienced a more prolonged or invasive course (e.g., requiring surgical completion of an incomplete abortion) still found the medical approach acceptable overall. This underscores that while efficacy largely drives satisfaction, other factors—such as the desire to avoid surgery and to undergo the process in a private or familiar setting—continue to make the medical method appealing to many patients.

From an emotional and psychological perspective, the timing of the two-step regimen may influence patient experience. Some patients find the 48-h waiting period between mifepristone and misoprostol to be emotionally challenging, as it prolongs the presence of an unwanted pregnancy and can heighten anxiety during the interim [37]. For some patients, a concurrent regimen could reduce this distress by shortening the process and providing faster resolution. However, this potential benefit must be carefully weighed against the disappointment and anxiety that can accompany treatment failure and subsequent need for surgical intervention.

The growing role of telemedicine in abortion care further complicates this balance. During the COVID-19 pandemic, many health systems expanded telehealth protocols that allowed patients to receive both mifepristone and misoprostol by mail and self-administer them at home under remote supervision [7,38]. In this context, a concurrent regimen appears particularly attractive, as it minimizes the need for repeat visits and enables a “one-stop” approach [39]. From a health system perspective, such a model could reduce clinic workload, improve efficiency, and potentially lower costs by consolidating care into a single encounter. On the other hand, any increase in treatment failures could offset these advantages by requiring more follow-up, additional appointments, or surgical procedures. Cost-effectiveness analyses have suggested that regimens with similar efficacy can differ in overall resource use depending on context, patient adherence, and healthcare infrastructure [40]. Thus, the optimal regimen may vary by setting.

In resource-limited or humanitarian environments, where repeated visits may be impossible, even a somewhat less effective regimen might be preferable if it ensures that more patients can access safe abortion earlier. A same-day regimen could help prevent delays that lead to second-trimester procedures, which are more complex and carry higher complication rates. Conversely, in high-resource settings with reliable follow-up, maximizing efficacy through a 48-h protocol may better serve both patients and health systems.

Overall, our trial reinforces that patient-centered care should remain the guiding principle. While the 48-h interval regimen continues to represent the gold standard due to superior efficacy, there are scenarios where concurrent administration may be considered appropriate. Shared decision-making is key: patients should be fully informed about the trade-off between convenience and efficacy, and their personal circumstances—such as travel barriers, childcare responsibilities, or limited clinic access—should be factored into the final choice. For some patients, the convenience and emotional relief of a same-day regimen may outweigh its lower efficacy, as long as appropriate follow-up and surgical backup are available.

Future studies should continue to explore the role of concurrent dosing in different care models. In telehealth settings, where patients may receive both medications by mail and self-administer at home, a simplified regimen could be appealing. Similarly, research in low- and middle-income countries is important, as limited access to follow-up care may alter the balance between convenience and efficacy. Finally, future evaluations should include cost-effectiveness analyses, since the overall value of a regimen depends not only on clinical outcomes but also on how it affects health system resources and the need for additional interventions.

### Strengths and Limitations

This study has several strengths. It is, to our knowledge, the first randomized controlled trial directly comparing concurrent dosing with the standard 48-h interval protocol using a fixed combination of 600 mg mifepristone and 400 mcg oral misoprostol. Its prospective design, standardized follow-up, and use of objective measures such as hemoglobin levels enhance the reliability of the findings. The involvement of a limited number of experienced clinicians minimized variability in counseling and assessment, further strengthening internal validity.

Nevertheless, limitations should be acknowledged. The regimen employed differs from the more commonly used combination of 200 mg mifepristone followed by 800 mcg misoprostol. Although rare, there are recent studies that have evaluated our regimen [41], supporting its relevance in certain clinical contexts. Our findings may therefore underestimate the efficacy of concurrent administration when higher misoprostol doses or alternative routes are used. Additionally, the relatively short follow-up period may have missed late expulsions or delayed complications. Finally, as this was a single-center study, results may not fully generalize to other populations or healthcare systems.

## 5. Conclusions

Concurrent administration of 600 mg mifepristone and 400 mcg oral misoprostol is associated with significantly lower efficacy than the 48-h interval regimen for early medical abortion. Although safety profiles were comparable, the reduced success and satisfaction rates suggest that concurrent administration should not replace the standard protocol. Nevertheless, concurrent dosing may still have a role in specific contexts where convenience or accessibility considerations are paramount. Patients considering same-day treatment should be counseled regarding the increased risk of treatment failure and the potential need for further intervention.

## Figures and Tables

**Table 1:** Demographic and obstetric variables of patients according to treatment group.

Characteristic	Concurrent Treatment (n = 124)	Control Group (n = 120)	*p*-Value
Age, mean ± SD (range)	31.9 ± 6.4 (20–46)	31.5 ± 6.4 (18–48)	0.65
BMI, mean ± SD (range)	24.2 ± 5.0 (16.9–46.9)	25.2 ± 5.1 (16.5–39.4)	0.14
Married, n (%)	92 (74.2)	91 (75.8)	0.77
Smoker, n (%)	20 (16.1)	16 (13.3)	0.54
Background disease ^1^, n (%)	26 (21.0)	25 (20.8)	0.98
Gravidity, mean ± SD (range)	4.2 ± 1.9 (1–10)	4.5 ± 2.3 (1–11)	0.20
Parity, mean ± SD (range)	2.2 ± 1.5 (0–6)	2.4 ± 1.7 (0–7)	0.19
Past ectopic pregnancy, n (%)	3 (4.0)	1 (1.2)	0.34
Hemoglobin at admission, median ± IQR (range)	12.4 ± 1.1 (9.5–14.5)	12.4 ± 1.4 (9.2–14.9)	0.26

^1^ Includes Diabetes Mellitus, Hypertension, Controlled Asthma, Hypothyroidism, Depression, Thrombophilia, Anemia, Essential Thrombocytosis, Familial Mediterranean Fever, Parkinson’s Disease, Epilepsy, Crohn’s disease, Rheumatoid Arthritis, Celiac, Fibromyalgia, Multiple Sclerosis, Migraine, Bell’s Palsy. Continuous variables are mean ± IQR; categorical variables are N (%).

**Table 2:** Current pregnancy data of patients according to treatment group.

Exam Data	Concurrent Treatment (n = 124)	Control Group (n = 120)	*p*-Value
Clinical size of uterus, mean ± SD (range)	6.0 ± 0.6 (5–7)	6.0 ± 0.6 (4–7)	0.68
Sonographic gestational age, mean ± SD (range)	6.0 ± 0.6 (4.5–7.0)	6.0 ± 0.6 (4.4–7.0)	0.76
Yolk sac present, n (%)	117 (94.4)	116 (96.7)	0.38
Fetal pole present, n (%)	103 (83.1)	96 (80.0)	0.54
Fetal pulse present, n (%)	93 (75.0)	88 (73.3)	0.77
Bleeding during current pregnancy, n (%)	10 (8.1)	12 (10.0)	0.60

Continuous variables are mean ± IQR; categorical variables are N (%).

**Table 3:** Outcome measures of patients according to treatment group.

Outcome Measure	Concurrent Group (n = 112)	Control Group (n = 108)	*p*-Value
Need for surgical intervention or misoprostol, n (%)	35 (31.2)	17 (15.7)	0.007
Need for surgical intervention, n (%)	31 (27.7)	16 (14.8)	0.02
Need for additional misoprostol dose, n (%)	5 (4.5)	1 (0.9)	0.11
Continuing pregnancy, n (%)	15 (13.4)	3 (2.8)	0.004
Any vaginal bleeding, n (%)	41 (36.6)	31 (28.7)	0.21
Post-abortion hemoglobin, median ± IQR (range)	12.3 ± 2.20 (4.5–14.1)	11.9 ± 1.73 (7.5–14.8)	0.35
Hemoglobin level decrease, median ± IQR (range)	0.20 ± 0.90 (0.0–8.7)	0.30 ± 0.83 (0.0–2.7)	0.61
VNRS pain score, median ± IQR (range)	5.0 ± 6.0 (0–10)	4.0 ± 4.0 (0–10)	0.33
Level of satisfaction, median ± IQR (range)	5.0 ± 4.0 (1–5)	5.0 ± 1.0 (1–5)	0.04
“Would you repeat?” Yes, n (%)	71 (72.4)	73 (82.0)	0.12

Continuous variables are mean ± IQR; categorical variables are N (%).

**Table 4:** Occurrence of adverse events in patients by treatment group.

Adverse Events	Concurrent Group (N = 112)	Control Group (N = 108)	*p*-Value
Excessive Bleeding	6 (5.4)	3 (2.8)	0.34
Emergency Surgical Intervention for Bleeding	6 (5.4)	3 (2.8)	0.34
Blood Transfusion	2 (1.8)	0 (0.0)	0.50
Pelvic Inflammatory Disease	1 (0.9)	1 (0.9)	>0.99
Hospital Admission	7 (6.3)	3 (2.8)	0.22
Hysteroscopy For Retained Product Of Conception	6 (5.4)	9 (8.3)	0.38

Categorical variables—N (%).

## Data Availability

The data from this study are available from the corresponding author upon a reasonable request and following approval of the institutional review board.

## Supplementary Materials

The following supporting information can be downloaded at: https://www.mdpi.com/article/10.3390/jcm14217616/s1, Satisfaction Survey Template; CONSORT 2025 Checklist [42].

## Abbreviations

The following abbreviations are used in this manuscript:

ACOG	American College of Obstetricians and Gynecologists
FIGO	International Federation of Gynecology and Obstetrics
WHO	World Health Organization
CI	Confidence Interval
IQR	Interquartile Range
SD	Standard Deviation
FDA	Food and Drug Administration
IRB	Institutional Review Board
IUD	Intrauterine Device
PID	Pelvic Inflammatory Disease
RPOC	Retained Products of Conception
TOP	Termination of Pregnancy
VNRS	Verbal Numerical Rating Scale
