# Supplementary material for: Early Pregnancy Termination with Mifepristone and Misoprostol: Concurrent vs. 48-Hour Interval Administration in a Randomized Controlled Trial
Source: J Clin Med. 2025 Oct 27;14(21):7616. doi: 10.3390/jcm14217616 (PMC12609839; doi:10.3390/jcm14217616)
Supplement: Supplementary file 1 [file jcm-14-07616-s001.zip › Patient_Satisfaction.pdf]

Patient Satisfaction Questionnaire

(Administered after completion of medical abortion treatment)

Study Title: Early Pregnancy Termination with Mifepristone and Misoprostol: Concurrent vs. 48-Hour Interval Administration
Participant ID: _____________ Date: _____________

Section A: Pain Assessment

1. Please rate the maximum pain you experienced during the process (circle one number):

0	1	2	3	4	5	6	7	8	9	10
No pain										Worst imaginable pain

Section B: Satisfaction with the Process

Please indicate your agreement with the following statement:
“The process was performed to my satisfaction.”

Response option	Please tick (✓)
1 – Strongly Disagree	
2 – Disagree	
3 – Neutral	
4 – Agree	
5 – Strongly Agree	

Section C: Future Preference

Would you choose to repeat this method of termination in the future if required?
■ Yes ■ No
